# Supplementary material for: A xanthene derivative, DS20060511, attenuates glucose intolerance by inducing skeletal muscle-specific GLUT4 translocation in mice
Source: Commun Biol. 2021 Aug 20;4:994. doi: 10.1038/s42003-021-02491-6 (PMC8379256; doi:10.1038/s42003-021-02491-6)
Supplement: Supplementary file 2 — Supplementary Information [file 42003_2021_2491_MOESM2_ESM.pdf]

Flow to DS20060511

Random screening using L6-GLUT4myc myotubes to identify compounds that would rapidly induce GLUT4 translocation in skeletal muscle cells

⇩ 2 compounds

Lactate production assay in L6-GLUT4myc myotubes as a counter assay to exclude compounds with toxic effects, such as respiratory chain inhibition

⇩ 2 compounds

2-DG uptake assay in L6-GLUT4myc myotubes to confirm increase of glucose uptake

⇩ 2 compounds

Akt phosphorylation assay in L6-GLUT4myc myotubes to exclude insulin signaling modulators

⇩ 1 compound

The original hit xanthene compound

⇩

Optimization of the hit compound through derivatization

⇩

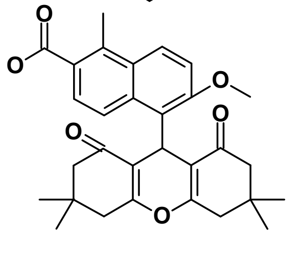

DS20060511

Supplementary Figure 1. Flow to the identification of DS20060511

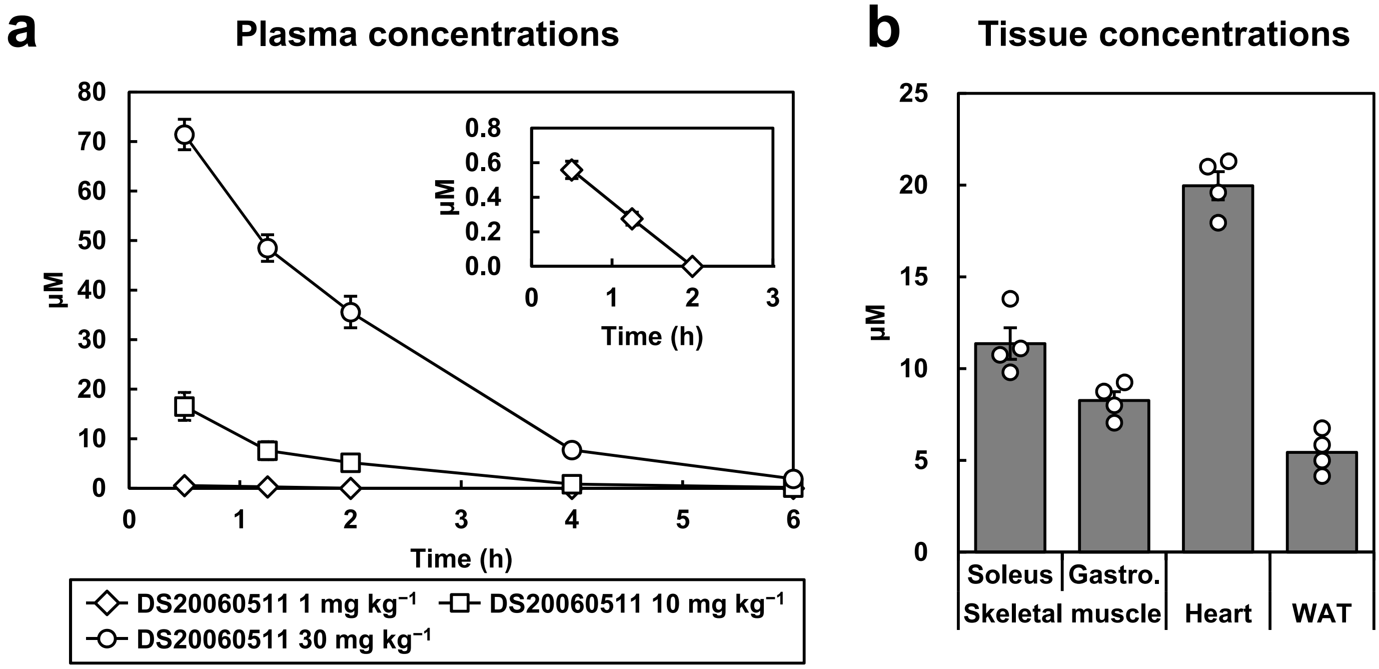

Supplementary Figure 2. Pharmacokinetics of DS20060511. a Plasma concentration changes of DS20060511 at the indicated timepoints after oral administration of 1, 10, and 30 mg kg^{-1} of DS20060511 in C57BL/6 mice (n = 4). **b** Tissue concentrations of DS20060511 in the soleus and gastrocnemius (Gastro) muscles, inguinal white adipose tissue (WAT), and liver of C57BL/6 mice 75 min after oral administration of 30 mg kg^{-1} of DS20060511 (n = 4). Tissue concentrations of the compound were calculated by assuming the volume of 1 g of tissue is 1 mL. Data are means ±SEM.

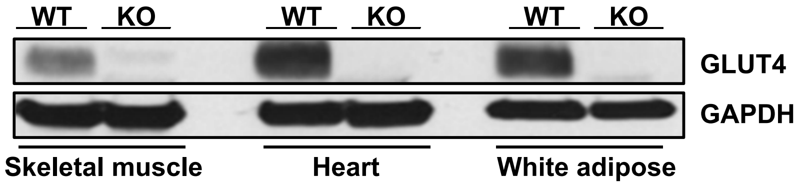

Supplementary Figure 3. GLUT4 protein levels are undetected in GLUT4KO mice. Protein levels of GLUT4 and GAPDH in skeletal muscle, heart and white adipose of WT and GLUT4KO mice.

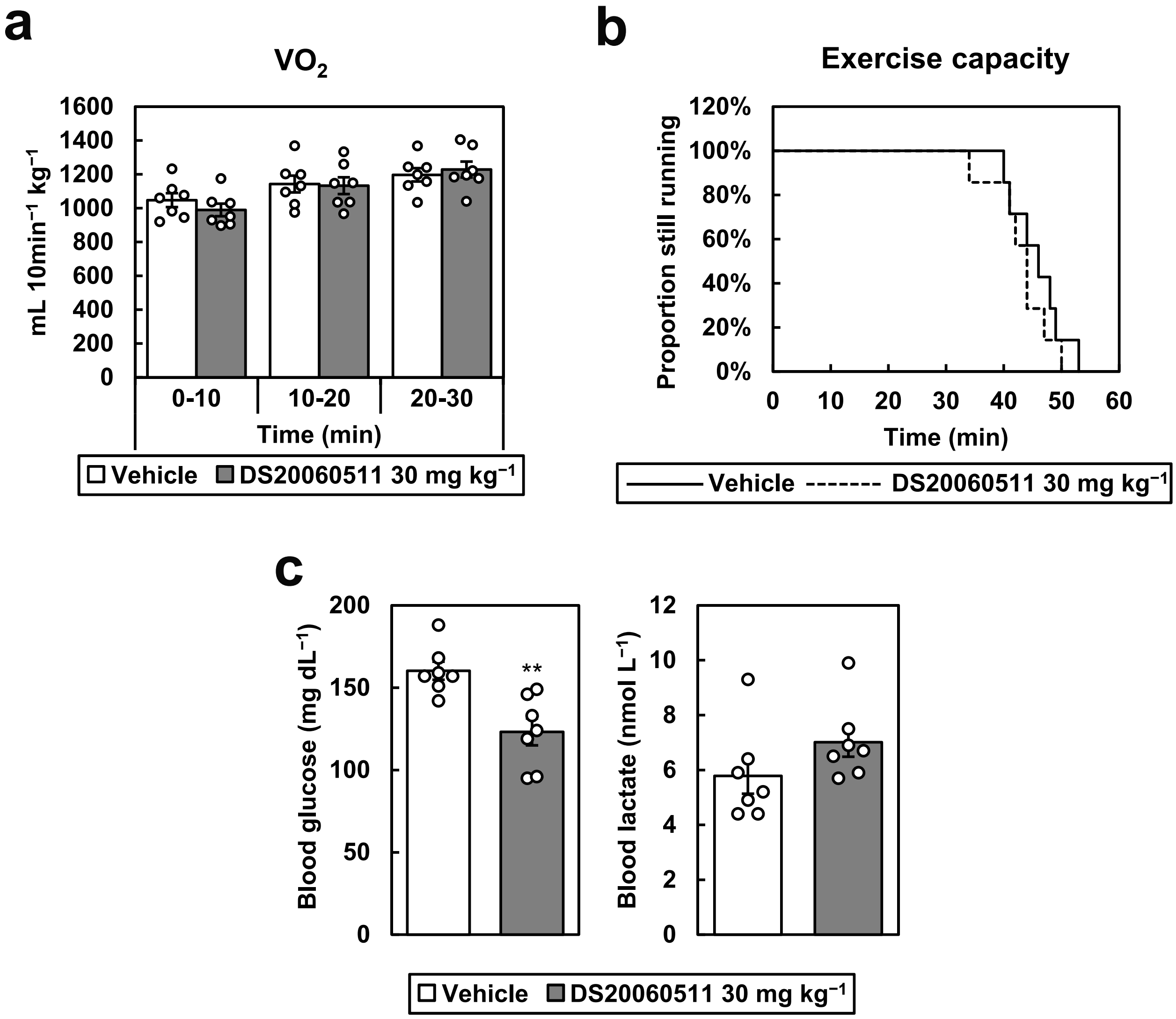

Supplementary Figure 4. Oxygen consumption, exercise capacity, and blood glucose and lactate levels after treadmill running. a VO_2 and **b** Exercise capacity during stepwise treadmill running in the C57BL/6 mice (n = 7). **c** Blood glucose and lactate levels after stepwise treadmill running exercise in the C57BL/6 mice (n = 7). Values shown are means ± SEM. **P<0.01 vs. vehicle by the t-test.

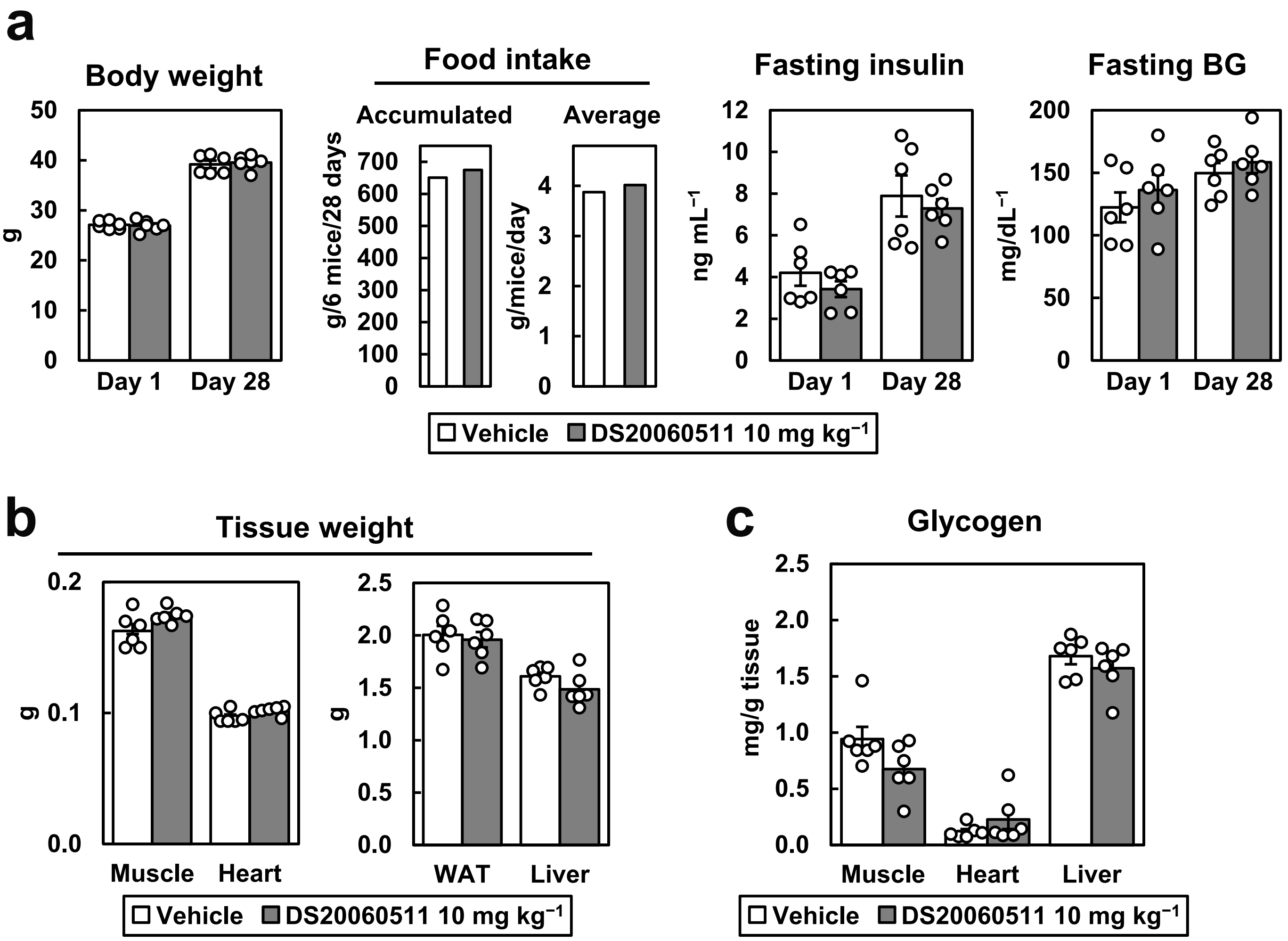

Supplementary Figure 5. No changes in the body weight, food intake, fasting insulin levels, blood glucose levels, tissue weight or glycogen content were observed in the DS20060511-treated *db/db* mice. a Body weight, food intake, fating insulin, and blood glucose levels measured before and after repeated treatment of DS20060511. **b** Tissues weights of the triceps surae muscle (muscle), heart, epididymal white adipose tissue (WAT), and liver collected from the *db/db* mice after 28-day repeated treatment with DS20060511 (n = 6). **c** Glycogen contents of the muscle, heart and liver (n = 6). No statistically significant differences were observed between the DS20060511-treated and vehicle-treated mice.

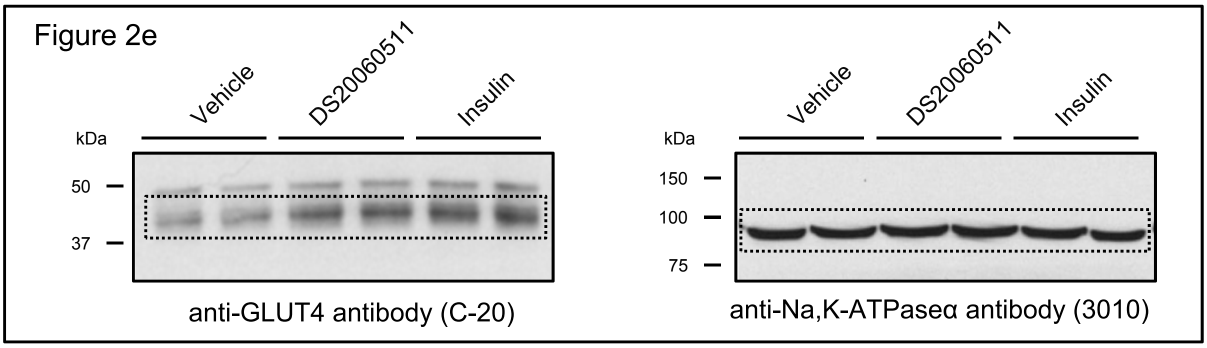

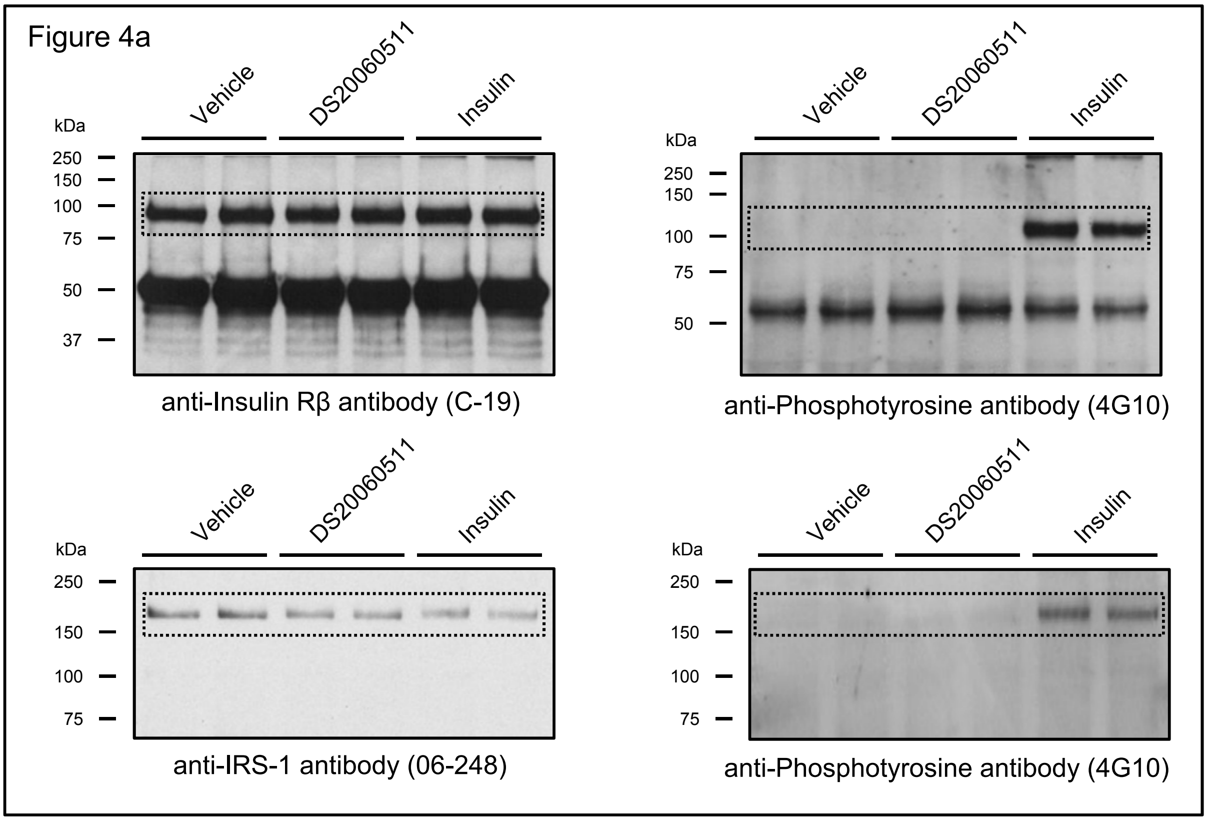

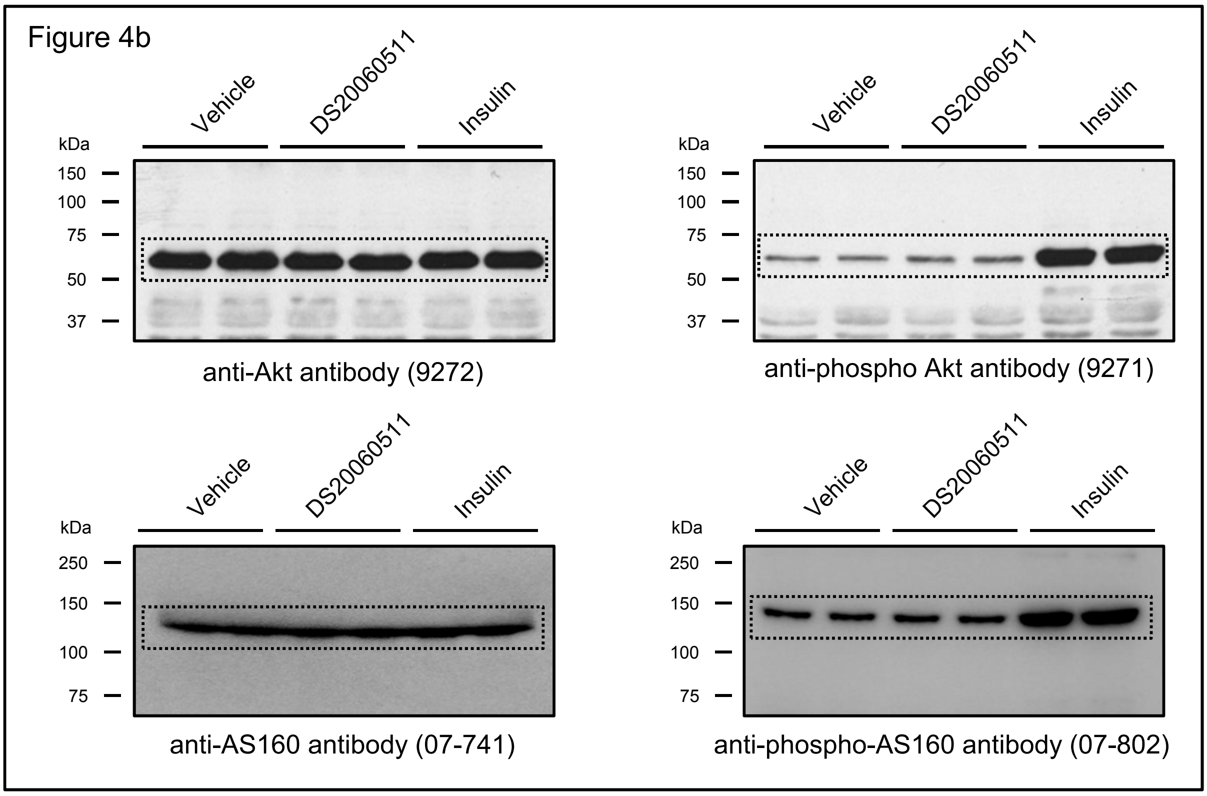

Supplementary Figure 6. Uncropped western blot images shown in Fig. 2e, Fig. 4a and Fig. 4b.

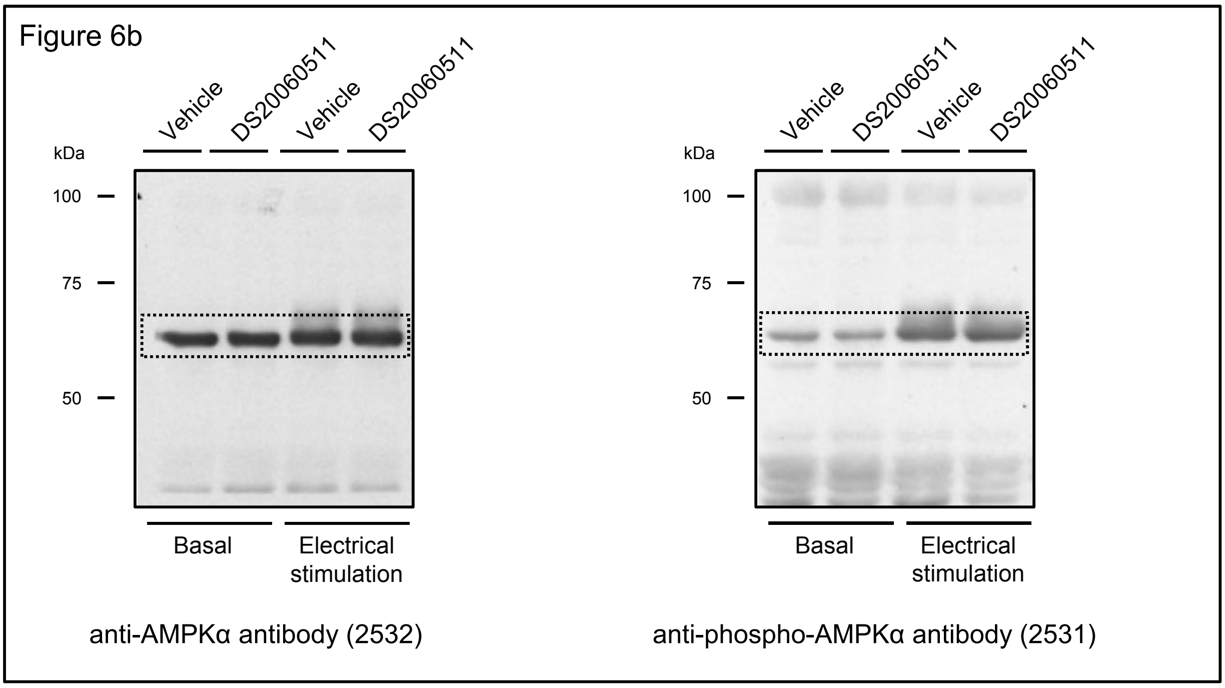

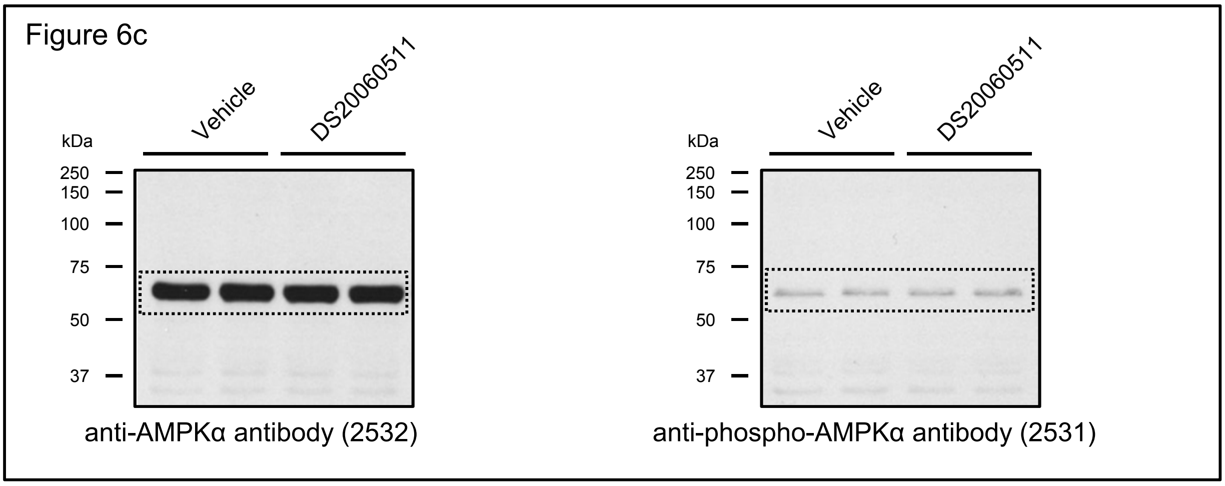

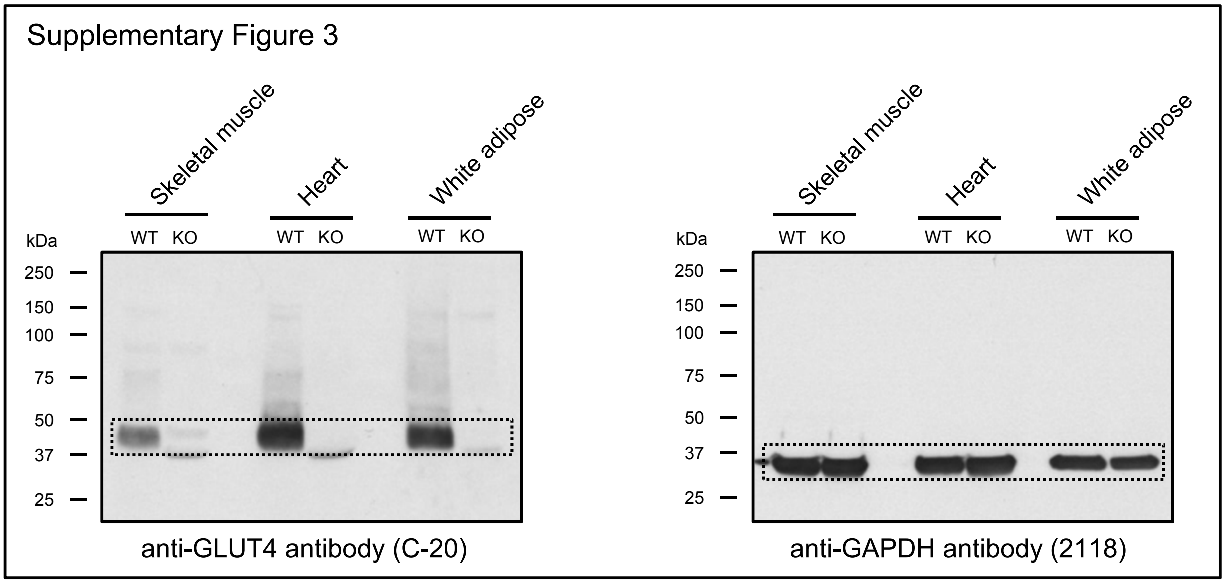

Supplementary Figure 7. Uncropped western blot images shown in Fig. 6b, Fig. 6c and Supplementary Fig. 3.
