# Supplementary material for: A xanthene derivative, DS20060511, attenuates glucose intolerance by inducing skeletal muscle-specific GLUT4 translocation in mice
Source: Commun Biol. 2021 Aug 20;4:994. doi: 10.1038/s42003-021-02491-6 (PMC8379256; doi:10.1038/s42003-021-02491-6)
Supplement: Supplementary file 4 — Reporting Summary [file 42003_2021_2491_MOESM4_ESM.pdf]

nature research

Corresponding author(s): Naoto Kubota

Last updated by author(s): Jul 15, 2021

Reporting Summary

Nature Research wishes to improve the reproducibility of the work that we publish. This form provides structure for consistency and transparency in reporting. For further information on Nature Research policies, see our Editorial Policies and the Editorial Policy Checklist.

Statistics

For all statistical analyses, confirm that the following items are present in the figure legend, table legend, main text, or Methods section.

n/a	Confirmed	
☐	☒	The exact sample size (*n*) for each experimental group/condition, given as a discrete number and unit of measurement
☐	☒	A statement on whether measurements were taken from distinct samples or whether the same sample was measured repeatedly
☐	☒	The statistical test(s) used AND whether they are one- or two-sided<br>*Only common tests should be described solely by name; describe more complex techniques in the Methods section.*
☒	☐	A description of all covariates tested
☒	☐	A description of any assumptions or corrections, such as tests of normality and adjustment for multiple comparisons
☐	☒	A full description of the statistical parameters including central tendency (e.g. means) or other basic estimates (e.g. regression coefficient) AND variation (e.g. standard deviation) or associated estimates of uncertainty (e.g. confidence intervals)
☒	☐	For null hypothesis testing, the test statistic (e.g. *F*, *t*, *r*) with confidence intervals, effect sizes, degrees of freedom and *P* value noted<br>*Give P values as exact values whenever suitable.*
☒	☐	For Bayesian analysis, information on the choice of priors and Markov chain Monte Carlo settings
☒	☐	For hierarchical and complex designs, identification of the appropriate level for tests and full reporting of outcomes
☒	☐	Estimates of effect sizes (e.g. Cohen's *d*, Pearson's *r*), indicating how they were calculated

Our web collection on statistics for biologists contains articles on many of the points above.

Software and code

Policy information about availability of computer code

Data collection	n/a
Data analysis	SAS System Release 9.2 (SAS Institute, Cary, NC)<br>GraphPad Prism 5.0 (GraphPad Software, San Diego, CA)<br>Excel 2013 (Microsoft, Redmond, WA)

For manuscripts utilizing custom algorithms or software that are central to the research but not yet described in published literature, software must be made available to editors and reviewers. We strongly encourage code deposition in a community repository (e.g. GitHub). See the Nature Research guidelines for submitting code & software for further information.

Data

Policy information about availability of data

All manuscripts must include a data availability statement. This statement should provide the following information, where applicable:

- Accession codes, unique identifiers, or web links for publicly available datasets
- A list of figures that have associated raw data
- A description of any restrictions on data availability

All data generated or analyzed in this this study are available within the article, its Supplementary Information file, and its Supplementary Data 1, or will be made available by the corresponding author upon request.

Field-specific reporting

Please select the one below that is the best fit for your research. If you are not sure, read the appropriate sections before making your selection.

☒ Life sciences ☐ Behavioural & social sciences ☐ Ecological, evolutionary & environmental sciences

For a reference copy of the document with all sections, see nature.com/documents/nr-reporting-summary-flat.pdf

Life sciences study design

All studies must disclose on these points even when the disclosure is negative.

Sample size	No statistical methods were used to predetermine sample size. The information on sample size for each experiment is provided in the figure legend.
Data exclusions	No data was excluded in this study.
Replication	All our results were obtained from distinct samples or independent experiments. Three biological replicates were used in each experiment, allowing data reproducibility. Non-numeric data are shown as representative results from more than three independent experiments.
Randomization	Male littermates were randomly and equally allocated to experimental groups.
Blinding	Not applicable.

Reporting for specific materials, systems and methods

We require information from authors about some types of materials, experimental systems and methods used in many studies. Here, indicate whether each material, system or method listed is relevant to your study. If you are not sure if a list item applies to your research, read the appropriate section before selecting a response.

Materials & experimental systems

n/a	Involved in the study	
☐	☒	Antibodies
☐	☒	Eukaryotic cell lines
☒	☐	Palaeontology and archaeology
☐	☒	Animals and other organisms
☒	☐	Human research participants
☒	☐	Clinical data
☒	☐	Dual use research of concern

Methods

n/a	Involved in the study	
☒	☐	ChIP-seq
☒	☐	Flow cytometry
☒	☐	MRI-based neuroimaging

Antibodies

Antibodies used	Antibodies against c-Myc (9E10, sc-40 and A-14, sc-789), Glut4 (C-20, sc-1608), and IRβ (C-19, sc-711) were purchased from Santa Cruz Biotechnology. Antibodies against Akt (9272), phospho-Akt (9271), AMPKα (2532), phospho-AMPKα (2531), Na,K-ATPaseα (3010), GAPDH (2118), anti-mouse IgG (7076), and anti-rabbit IgG (7074) were purchased from Cell Signaling Technology, Danvers, MA. Rabbit polyclonal antibody against IRS1 (06-248), mouse monoclonal antibody against phosphotyrosine (05-321), rabbit antiserum against AS160 (07-741), and rabbit polyclonal antibody against phospho-AS160 (07-802) were purchased from Merck Millipore. HRP-conjugated donkey anti-goat IgG (V8051) was purchased from Promega Corporation, Madison, WI. HRP-conjugated goat anti-rabbit IgG (111-035-144) and goat anti-mouse IgG (115-035-003) were purchased from Jackson ImmunoResearch, Laboratories, West Grove, PA.
Validation	All antibodies used in this study were validated by the suppliers.

Eukaryotic cell lines

Policy information about cell lines

Cell line source(s)	L6-GLUT4myc rat myoblast cell line, 3T3-L1-GLUT4myc fibroblast cell line
Authentication	The L6-GLUT4myc rat myoblast cell line was obtained from Dr. Amira Klip and Dr. Philip Bilan, through a license granted by The Hospital for Sick Children, 555 University Avenue, Toronto, Ontario, Canada M5G 1X8; or purchased from Kerafast, Boston, MA (ESK202). The 3T3-L1-GLUT4myc fibroblast cell line was provided by Dr. Tomoyuki Yuasa, Tokushima University.
Mycoplasma contamination	The cell lines were not tested for Mycoplasma contamination.

Commonly misidentified lines (See ICLAC register)	There was no commonly misidentified cell lines used in this study.

Animals and other organisms

Policy information about studies involving animals; ARRIVE guidelines recommended for reporting animal research

Laboratory animals	Male mice (8-12 weeks old) were used. C57BL/6 and db/db mice. GLUT4 and eNOS knockout mice.
Wild animals	Not used
Field-collected samples	Not used
Ethics oversight	The animal care and experimental procedures used in the study were approved by The University of Tokyo Animal Care Committee (Approval number: 27-3), and the study was carried out in accordance with the Animal Experimentation Guidelines of Daiichi-Sankyo Co., Ltd (Approved number: 20000411).

Note that full information on the approval of the study protocol must also be provided in the manuscript.
