# Supplementary material for: A xanthene derivative, DS20060511, attenuates glucose intolerance by inducing skeletal muscle-specific GLUT4 translocation in mice
Source: Commun Biol. 2021 Aug 20;4:994. doi: 10.1038/s42003-021-02491-6 (PMC8379256; doi:10.1038/s42003-021-02491-6)
Supplement: Supplementary file 5 — Description of Supplementary Files [file 42003_2021_2491_MOESM5_ESM.pdf]

Description of Additional Supplementary Files

File name: Supplementary Data 1

Description: Source data for the graphs and charts in main and supplementary figures.
